# Therapeutic approaches for anti-sperm-antibodies in the testicular sperm aspiration rat model

**DOI:** 10.14202/vetworld.2023.296-308

**Published:** 2023-02-15

**Authors:** Abdel-Kader A. Zaki, Fahad S. Aldahmashi, Abd El-Nasser A. Madboli, Kamal A. Attia, Fahad S. Almulhim, Saleh M. Albarrak

**Affiliations:** 1 Department of Veterinary Medicine, College of Agriculture and Veterinary Medicine, Qassim University, Buraydah, Saudi Arabia; 2 Department of Physiology, Faculty of Veterinary Medicine, Cairo University, Giza, Egypt; 3 Ministry of Environment, Water and Agriculture, Qassim Region, Saudi Arabia; 4 Department of Animal Reproduction and Artificial Insemination, National Research Center, Veterinary Research Institute, Giza, Egypt; 5 Department of Biology, Al-Jammoum University College, Umm-Alqura University, Makkah, Saudi Arabia

**Keywords:** anti-sperm antibody, enzyme-linked immunosorbent assay, infertility, testicular sperm aspiration, testosterone

## Abstract

**Background and Aim:**

Anti-sperm antibodies (ASAs) treatment continued to be neglected. This study aimed to generate ASAs using the testicular sperm aspiration (TSA) rat model, which allowed for investigation of four distinct therapeutic approaches to find potential treatments for ASAs.

**Materials and Methods:**

Adult Wistar albino male rats were divided into six equal groups (n = 12). The negative control group underwent scrotal sac surgery without having their testicles punctured. Punctures were made in the remaining 5 groups, with one group left untreated to serve as the positive control group. The remaining 4 groups were treated with either dexamethasone (DEX), azathioprine (AZA), frankincense, or anti-ASAs secondary antibodies. For 10 weeks, serum samples were collected every 2 weeks for specific quantification of ASAs. Testis and epididymis tissues were collected for histopathological analysis.

**Results:**

The ASAs concentrations of the positive controls were significantly higher (p ≤ 0.001) than their negative control counterparts during the examined weeks. However, The ASAs indices (%) differed according to the treatment type. While the ASAs indices at the 2^nd^ and 4^th^ weeks in the AZA-treated group were significantly reduced compared to the positive control group (p ≤ 0.001), no significant differences were observed at any of the sample collection week for the DEX-treated rats. The ASAs indices were significantly decreased only at weeks 6 and 8 of treatment in the frankincense-treated group (p ≤ 0.001). In the secondary antibodies-treated group, the antibody indices were significantly decreased in all weeks except for samples collected at week 4 (p ≤ 0.001). The testosterone levels reverted to normal only in TSA rats treated with either Frankincense or secondary antibodies, as they were significantly higher than the positive controls (p ≤ 0.05). Tissue samples from the secondary antibody-treated rats showed a generally normal histological appearance.

**Conclusion:**

This study tried to offer realistic therapy suggestions; however, caution should be applied when extrapolating findings from experimental models to meet clinical requirements.

## Introduction

Immunological male infertility can be caused by anti-sperm antibodies (ASAs); however, the exact mechanism is still unknown. It is well known that the blood-testis barrier in the testicle is composed of tightly packed cell connections that block immune cells from entering the lumen of the seminiferous tubules. The development of ASAs is likely caused by traumatic disturbance or developmental problems with the blood-testicular barrier that separates the sperm antigens from the immune system. Sperm cells, therefore, display novel antigens that the immune system has not previously encountered [1]. In males, autoimmune orchitis, traumatic conditions, and blood-testicular barrier abnormalities can result in ASAs generation [2]. The ASAs have a detrimental effect on sperm activity, quantity, and motility in humans and multiple veterinary species, including bulls, stallions, and dogs [3–8]. According to Lu *et al*. [8], ASAs have been detected in 5%–15% of infertile males, suggesting that they may contribute to male infertility in humans. Anti-sperm antibodies can be detected on the sperm surface and in seminal plasma and serum samples [3]. It has been demonstrated that sperm aggregation is related to immunoglobulin (Ig)A and IgG presence in the semen [5]. According to Onemu *et al*. [9], immunological infertility caused by ASAs affects the fertilization process and lowers the likelihood of becoming pregnant.

In general, autoimmune diseases are conventionally treated by the administration of immunosuppressive drugs [10]. Treatment with low doses of corticosteroids has reportedly been used to treat males with ASAs [11, 12]. There have been conflicting studies favoring and opposing treating ASAs with corticosteroids, leaving specialists in continual debate. In this study, we examined treating ASAs with corticosteroids (dexamethasone [DEX]), azathioprine (AZA), frankincense, and anti-ASAs secondary antibodies. Azathioprine is utilized as an immunosuppressant and a steroid maintenance drug, as demonstrated by Chavez-Alvarez *et al*. [13]. Azathioprine reduces DNA synthesis in highly proliferative cells such as T and B lymphocytes. Frankincense is oleogum resins, collected from a thick tree of the genus *Boswellia*; these trees are found in arid regions such as the Middle East, Africa, and India [14]. It has been used as an anti-inflammatory drug [15]. Production of therapeutic antibodies specific to other antibodies is known as anti-idiotype responses, presumably because the idiotype component is the primary “foreign” component of the inoculated antibody molecule [16]. Anti-idiotypic antibodies are now being researched as potential novel therapeutics for cancer, autoimmune disorders, and a few other diseases. In their review, Stanova *et al*. [17] suggested that secondary antibodies (Anti-idiotypic antibodies) might be able to specifically outcompete an antigen for binding to an antibody.

Testicular sperm aspiration (TSA) has been used to obtain sperm in various species using various gauge needle sizes, including humans, rams, and rats [18–20]. ASAs of the IgG isotype were detected in male sera following sperm extraction [21]. Humans are at risk of postsurgical fibrosis, and testosterone levels have been shown to fall following TSA [22, 23].

Despite substantial research on immunologically driven infertility, ASAs testing and treating ASAs-positive cases continued to be neglected. Many laboratories no longer perform ASAs testing and advise against using ASAs readings during the initial stage of male examination. There is little research to back up the use of steroids as a treatment for immunological infertility, and adverse effects from extended or high-dose steroid therapy have raised concerns. This disparity emphasizes the need for more research to fully comprehend the effects of ASAs testing and treatment.

Therefore, TSA as a model of male albino rats was used in this study to generate ASAs to investigate four different therapeutic approaches to obtain possible solutions for ASAs treatment. This study highlighted how ASAs cause infertility by reducing the quality of sperm and the rate of conception due to low and slowing sperm motility.

## Materials and Methods

### Ethical approval

The study was approved by Animal Care and Use Committee of the Experimental Animal Centre at Qassim University (Approval Number: 23-24-20). The anesthetic used for all surgeries was diethyl ether, and every effort was made to lessen the pain.

### Study period and location

The study was conducted from August 2020 to March 2021. The study was conducted at the Department of Veterinary Medicine, College of Agriculture and Veterinary Medicine, Qassim University, Saudi Arabia.

### Animals

Seventy-two adult Wistar albino male rats with 180–200 g average body weight, procured from the experimental animal unit, College of Pharmacy, King Saud University, Riyadh city, Saudi Arabia., were divided into six groups (n = 6/cage) and housed in an environmentally controlled room (22°C ± 2°C/40%–60% RH). Tap water and standard chow were available to rats as 35% carbohydrates, 25% protein, 7% lipids, and 3% vitamins. Twelve-hour light-dark cycles were used for the whole trial as well as the week prior. Male rats were prepared for testicular surgery at room temperature (25°C).

### Testicular sperm aspiration procedure and treatment groups

Each rat was placed in a vacuum-sealed container and anesthetized by diethyl ether inhalation. The rat was put on its back after being given the anesthetic and the scrotum was tightened to allow for testicular protrusion. The scrotum was then opened between the testicles while holding the testicles between the index finger and thumb. The testicles were then taken out and holes were cut in the left testicle. The testicle contents were extracted with a needle, and a simple suture was performed (TSA). Baneocin antibiotic powder (Sandoz Co. Kundl, Tirol, 6250 Austria) was applied to prevent infection. Twelve rats had scrotal sac surgery without having their testicles punctured, and these animals served as the negative control group. Testicular sperm aspiration rats were divided into five groups (n = 12) in separate cages. In the positive control group, the testicular puncture was performed on day 0 of the experiment, and rats were left without any medicinal intervention. The dexamethasone-treated group was injected intramuscularly with DEX sodium phosphate 14 days post testicular puncture using the regime of 2.25 mg/kg body weight/twice weekly/3 weeks [24]. The DEX concentration used was 8 mg/2 mL/EPICO.

Azathioprine-treated group: The AZA drug was obtained as Imuran 50 mg tablets from a pharmacy (Sitcopharma, Aspen Pharma Trading Limited Co., Germany). Only 40 mg of each tablet, which was powdered, was used. The 40 mg powder was then diluted in 10 mL normal saline solution (Sodium chloride 0.9% solution) and used at a therapeutic dosage of 20 mg/kg body weight every day for 2 weeks, according to Onanuga *et al*. [25]. The treatment was administrated orally through a stomach tube. Frankincense-treated group: Frankincense male gum was collected from Hojari, Salalah, Sultanate of Oman [26]. A final concentration of 1 g/30 mL distilled water was achieved by dissolving the raw material in distal water after it had been crushed in a mortar. For 30 days, 100 mg/daily was given to each rat. Frankincense male gum was given orally to rats through a stomach tube. Secondary antibodies-treated group: This group received an intraperitoneal injection of secondary antibodies (after potency was determined, as will be detailed later) 14 days after the testicular puncture. The treatment dose was 50 mg/kg body weight twice weekly for 3 consecutive weeks.

### Blood sampling

The blood samples (3 mL/animal) were taken from the eye inner canthus of rats before performing the operations for the ASAs examination on day 0. Blood samples were collected every 2 weeks till the end of the experiment (10 weeks) for serum extraction.

### Testosterone levels

The testosterone levels were measured using a commercial enzyme-linked immunosorbent assay (ELISA) kit according to the manufacturer’s specifications (Monocent, Inc.’s Testosterone ELISA test system kits. REF. EL1-1263. 9025 Eton Ave. Ste C, Canoga Park, CA 91304, USA).

### Histopathological examination

Bancroft and Gamble’s histopathology methodology was followed Bancroft, J.D. and Gamble [27]. Following postmortem examination, tissue samples from the testis, epididymis, and spleen of all rat groups were collected and fixed using 10% neutral buffered formalin.

### Preparation of ASAs in rabbits

For pooled hyperimmune serum production, sperm proteins were administered to three male rabbits. As previously reported, sperm proteins were derived from corpus and testis semen [28]. The corpus content was placed in a microcentrifuge tube and allowed to liquefy for 30 min at room temperature (25°C). The epididymal seminal plasma and sperm were separated by centrifugation at 1500× *g* for 15 min at 4°C. The seminiferous tubules were taken out and diced in a phosphate buffer saline (PBS)-filled Petri plate (pH 7.4). After allowing the sperm to separate from the tubules, each aliquot was aspirated using a Pasteur pipette, resuspended in 1 mL of PBS (pH 7.4), and centrifuged at 1500× *g* at 4°C. The Testicular seminal plasma was considered to make up the remaining fluid. After repeating the washing process 3 times, sperm homogenization was done in a solution composed of 0.25 mol/L sucrose, 1 mmol/L ethylenediaminetetraacetic acid, and 0.05 mol/L Tris hydrochloride (pH 7.4) using a homogenizer (Staufen, Germany) at 0°C–4°C. The homogenate was then sonicated for 30 s at 10 kHz on ice [29]. The sonicates were centrifuged for 90 min at 8500× *g* at 4°C, and the concentration of the total protein in the supernatants was quantified using a colorimetric method (Biuret reagent) according to Doumas *et al*. [30]. The sperm were emulsified after an equivalent volume of Freund’s complete adjuvant was added (Difco, USA). Each animal received a 0.5 mL intra-peritoneal injection of the homogenate, which comprised 400 mg of sperm total proteins. Boosters were administered 2, 4, and 6 weeks following the first inoculation. A cardiac puncture was performed to collect blood 10 days following the fourth immunization. Sera were separated, pooled, and frozen at −20°C in aliquots. This pooled hyperimmune serum was utilized for the generation of the secondary antibodies as well as for the titration of their potency as a positive control in the ELISA assay.

### Immunoglobulin purification

According to a previous description by Abd El Hafez *et al*. [31], immunoglobulin precipitation was carried out using an ammonium sulfate solution. The serum was briefly placed into a flask and gently mixed to prevent foaming with continuous stirring. To achieve maximal precipitation and prevent mechanical entrapment of serum components other than gamma globulin in the precipitate, an equal amount of saturated ammonium sulfate (50%) was added drop by drop to the serum. The mixture was centrifuged at 1500× *g* for 20 min, and the supernatant was then discarded. The precipitate, which includes all of the gamma globulins, was then dissolved in PBS pH 7.2 to a final volume that was half or less than the original serum sample. Small molecular contaminants were eliminated using dialysis. The collected immunoglobulin precipitate was placed in a dialysis bag after being prepared per the manufacturer’s instructions (Sigma-Aldrich). Dialyzing against 15 mM PBS was carried out for 3 days at 4°C, with the PBS replaced each morning and evening to guarantee the removal of the ammonium sulfate. After being dialyzed, the immunoglobulins were taken out of the dialysis bag and centrifuged for 20 min at 1500× *g*. The supernatant contained the immunoglobulins that were separated into appropriate portions and stored in tubes at −20°C until needed. The levels of immunoglobulin solution were concentrated by polyethylene glycol by lowering its water content.

### Preparation of secondary antibodies

Animal immunization was carried out according to Muro *et al*. [32]. Rabbit IgG was given subcutaneously at a dosage of 40 mg IgG/kg body weight after being well mixed with an equivalent amount of complete Freund’s adjuvant (Difco). The same vaccination procedure was repeated 2 weeks post the initial injection. Booster doses were administered at 21 and 28 days of the first inoculation without the adjuvant. Before the immunization started, the first blood sample was collected to create a negative control for subsequent testing. The serum was then separated from blood samples that were collected 3 days after the last inoculation. After the rabbits were euthanized, 150 mL of blood from each one was taken, allowed to clot, and the separated serum was stored at −20°C until needed. Using the Doumas *et al*. [30] method, samples of rabbit serum were precipitated, dialyzed, concentrated, and their protein concentrations were assessed.

### Assessment of ASAs levels in rats’ serum

Levels of immunoreactive ASAs were determined using indirect ELISA according to the modified technique of standard indirect solid-phase ELISA that was reported by Esmailnejad *et al*. [7].

### Titration of sheep anti-rabbit IgG and sheep anti-rat IgG conjugated each with horseradish peroxidase (HRP)

Two ELISA microtiter – plates were coated with double-fold serial dilutions of sheep anti-rabbit IgG (product no. A-5279, Sigma Co., St. Louis, USA) and sheep anti-rat IgG (product no. A-5287, Sigma Co., St. Louis, USA) starting with the dilution of 1/10. This was accomplished by transferring 25 µL from a well into the next that contained 25 µL of blocking buffer (Bovine serum albumin 2% PH 7.2). An additional drop of 25 µL of the substrate solution was added to each well, followed by placing the plates in the incubator at 37°C for 30 min. The best dilutions were the highest concentration which gave an apparent positive reaction. The best dilutions were 1:4000 and 1:2000 for sheep anti-rabbit IgG and sheep anti-rat IgG, respectively.

### Determination of checkerboard titration

Checkerboard titration was performed to find the optimum dilution of the antigen and test the antisera to know the potency of the sperm protein antisera prepared in the rabbits. Serial dilutions of the tested antigen were used to coat the ELISA plate at 2000, 1000, 500, 250, 125, 62.5, and 31.25 ng, and a negative control antigen versus different dilutions of tested anti-sera diluted at 1:1, 1:2, 1:4, 1:8, 1:16, 1:32, and 1:64. The serum dilution with the lowest concentration of antigen/well that gave a clear signal (under the known dilution of conjugate and substrate) was considered the optimum condition and used in further tests. The potency of the rabbit antisera against the antigen (250 ng of sperm protein) was examined, and the antisera optimum dilution was 1:32.

### Standard log - dose response curve of sperm protein to determine the volume of sperm protein used

Serial dilutions of sperm proteins were placed in the plate (dilution/row) in the following order 250, 125, 62.5, 31.25, and 15.62 ng, followed by the negative control antigen. After incubating for 1 h at 37°C with shaking, the plate was washed 3 times with the washing buffer, loaded with 100 µL/well of the rabbit antisera (1:32 in PBS), and incubated for 1 h at 37°C with shaking. After incubation, the plate was washed 3 times, and a volume of 100 µL/well of the HRP-conjugated sheep anti-rabbit IgG (1:4000 dilution in PBS, PH 7.2) was added and incubated for 1 h at 37°C in a shaking water bath. After incubation, the plate was washed 3 times, and 100 µL/well of the substrate solution was added to each well. The reaction coloration was detected after incubating the plate in the dark for 7 min at room temperature. The reaction was then stopped by the addition of 50 µL/well of the stopping solution. The enzyme-mediated signal was measured at a wavelength of 492 nm using a microplate ELISA reader. A standard curve was then constructed, where optical density (OD) was plotted against the log dose of antigen.

### Standard log - dose response curve of secondary antibodies as antigen

Similar steps were done where serial dilutions of secondary antibodies instead of sperm proteins were placed in the plate (dilution/row) in the following order 5.61, 2.81, 1.40, and 0.70 µg, followed by the negative control. A standard curve was then constructed, where OD was plotted against the log dose of antigen. The levels of immunoreactive ASAs were determined using an indirect ELISA according to a modified technique of the standard indirect solid phase ELISA. Enzyme-linked immunosorbent assay is a diagnostic method frequently used in biological research to identify antibodies relating to a particular antigen by providing absorbance readings. A cutoff value was determined using known negative sera mixed with the unidentified samples in the microtiter plates to distinguish between positive and negative signals. The following is a general formula for a cutoff value: Cut-off = a*X + f * SD. Where X is the mean and SD is the standard deviation of independent negative control readings, and a and f are two multipliers. The value that discriminated between positive and negative samples was found and detected using change-point algorithms. The antibody indices (%) for each serum sample were calculated by comparison to positive and negative control values. The sensitivity% and specificity% were also computed.

### Analysis of sperm protein antisera in the serum of rats

The microplate was taken after incubation overnight at 4°C and wells were coated with 100 µL/well of rat sperm proteins (250 µg), diluted in carbonate bicarbonate coating buffer (pH 9.6), and incubated for 2 h at 37°C in a shaking water bath to distribute the protein in the wells. An adhesive tape was used to protect the plates from evaporation, and they were incubated at 4°C overnight to allow the solid phase to adsorb completely. Three times washings with the washing buffer (PBS pH 7.2 containing 0.05% Tween-20) eliminated any excess unbound sperm proteins. The remaining binding sites were blocked with 200 µL/well of blocking buffer, and the plates were incubated for 2 h at 37°C while being shaken. After rinsing 3 times with the washing buffer, each well received 100 µL of serially diluted rat serum samples, covered with an adhesive tape, and incubated for an hour at 37°C with shaking. The washing procedure was repeated, and 100 µL/well of the HRP-conjugated sheep anti-rat IgG (1:2000 dilutions in PBS, PH7.2) was added to each well and incubated for an hour at 37°C in a shaking water bath. Following the washing step, 100 uL/well of the substrate solution (TMB Innovative Diagnostics) was added to each well. The reaction was then stopped using the stopping solution (50 L/well) after the plates had been incubated in the dark for 7 min at room temperature. Using a microplate ELISA reader, the enzyme-mediated signal was detected at 492 nm wavelength.

### Statistical analysis

Values of data were illustrated as means ± standard errors. Analysis of variance was performed for statistical analysis, followed by Duncan’s Multiple range test, with p ≤ 0.05 being considered statistically significant. Statistical analysis was conducted with the Statistical Analysis System (SAS) program 21(SAS, USA).

## Results

### Anti-sperm antibodies levels assessment

**Figure-1:**
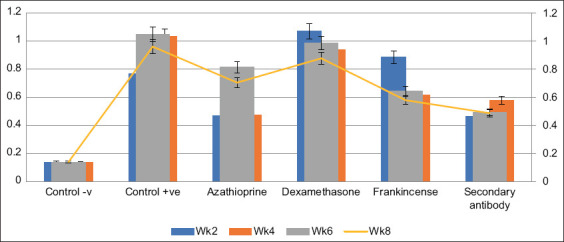
Optical density of samples at first dilution (1:1).

**Figure-2:**
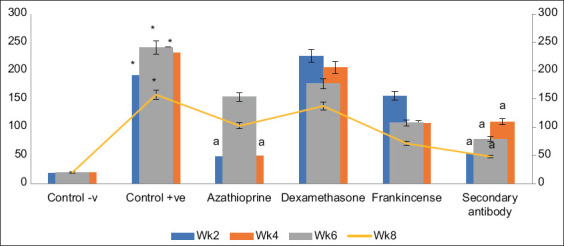
Concentration of anti-sperm antibody in samples at first dilution (ng/100 µL). Columns of positive control with * are significantly different than negative control at p < 0.001. Columns of treated groups with “a” are significantly different than positive control of the same week at p < 0.001, respectively.

The mean OD of the cutoff values of the rat sera was 0.0399 ± 0.0065 with an ASAs concentration of 12.35 ± 0.854 ng/100 µL. However, the dilution point (antibody titer) at which the cutoff value was reached, and antibody indexes (%) differed according to the treatment. The mean OD of the cutoff values of the negative control TSA rats at the 1/2 serial dilution were measured at all weeks, with the overall mean antibody index (%) being 17.355 ± 2.59. The results showed that the ASAs titer of the positive control TSA rats was 1/512 at week 2, 1/64 at week 4, 1/64, and 1/128 at the 6^th^ and 8^th^ weeks, with the overall mean antibody index (%) being117.365 ± 24.97. The findings revealed that the negative control TSA rats’ OD at the first dilution (1/1) was 0.1385 ± 0.0005 (Figure-1) with an ASAs concentration of 19.450.45 ng/100l in the 2^nd^ week, while at the 4^th^ week was 0.137 ± 0.0 with an ASAs concentration of 19.5 ± 0.0 ng/100 µL. The data also showed that the OD at first dilution (1/1) in the 6^th^ week was 0.1385 ± 0.0015 and the ASAs concentration was 19.95 ± 0.45 ng/100 µL (Figure-2). Furthermore, in the 8^th^ week, the OD at the first dilution (1/1) was 0.1405 ± 0.0035 with an ASAs concentration of 20.25 ± 1.65 ng/100 µL.

The analysis indicated that at week 2, the positive control TSA rats’ OD at the first dilution (1/1) was 0.77 ± 0.31, and their ASAs concentration was 191.75 ± 13.925 ng/100 µL. At week 4, however, the OD at the first dilution (1/1) was 1.0365 ± 0.0005, and the ASAs concentration was 331 ± 0.00 ng/100 µL. The data also showed that the OD at first dilution (1/1) at week 6 was 1.049 ± 0.011 with an ASAs concentration of 341 ± 0.00 ng/100 µL. Furthermore, at week 8, the OD at the first dilution (1/1) was 0.961 ± 0.119 with an ASAs concentration of 257.5 ± 80.5 ng/100 µL. All the values of the ASAs concentration of the positive controls were significantly higher (p ≤ 0.001) than their negative control counterparts at all the examined weeks.

**Figure-3:**
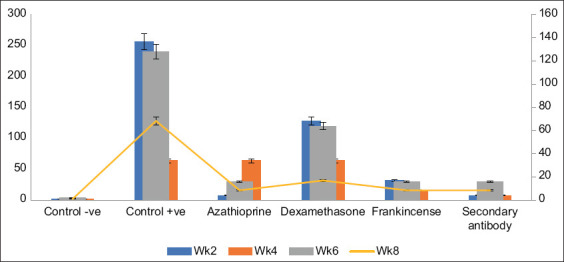
Antibody titers of measured samples at cutoff values.

**Figure-4:**
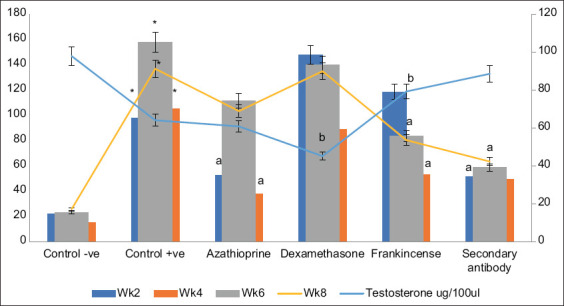
Antibody index (%) and testosterone levels (µg/100 µL). Columns of positive control with * are significantly different than negative control at p < 0.001. Columns of treated groups with a and b are significantly different than positive control of the same week at p < 0.001 and p < 0.05, respectively.

The ODs of the cutoff values of AZA -treated TSA rats were measured. The results showed that the cutoff value OD of the AZA-treated TSA group was observed at the 1/8 and 1/64 serial dilutions for weeks 2 and 4, respectively (Figure-3). Moreover, at weeks 6 and 8, it was detected at the serial dilution of 1/16. The results have also shown that the OD at first dilution (1/1) was 0.47 ± 0.095 in the 2^nd^ week and 0.4750 ± 0.085 in the 4^th^ week with ASAs concentrations of 48 ± 8.2 and 49.2 ± 4.5 ng/100 µL, respectively, both were significantly lower than the positive control group (p ≤ 0.001). In addition, the results revealed that at weeks 6 and 8, the OD at the first dilution (1/1) was 0.815 ± 0.055 and 0.705 ± 0.145 with ASAs concentrations of 153.5 ± 12.5 and 102.35 ± 48.56ng/100 µL, respectively. The mean values of ASAs index (%) at the 2^nd^ and 4^th^ week were 52.494 ± 10.02 and 37.576 ± 9.43, respectively, significantly reduced compared to the positive control group (p ≤ 0.001). However, the ASAs indexes tend to be higher in the 6^th^ and 8^th^ weeks (Figure-4).

The results demonstrated that at week 2, serial dilution 1/128 was the point at which the ODs of the cutoff value were detected in the serum samples of DEX -treated TSA rats. The cutoff value for the sera samples of the DEX -treated TSA rats was noted at the 1/64 serial dilution for the 4^th^ and 6^th^ weeks and at the 1/32 serial dilution for the 8^th^ week of treatment. The results showed that in the DEX -treated TSA rats, the OD at first dilution (1/1) at week 2 was 1.07 ± 0.01 with an ASAs concentration of 226.0 ± 3.0 ng/100 µL. However, at week 4, the OD at first dilution (1/1) was 0.938 ± 0.098, and the ASAs concentration was 205.5 ± 39.5 ng/100 µL. In addition, the results revealed that at week 6, the OD at first dilution (1/1) was 0.987 ± 0.0505, with the ASAs concentration being 177.0 ± 36.0 ng/100 µL. Furthermore, at week 8, the OD at first dilution (1/1) was 0.878 ± 0.42 with 137.5 ± 6.5 ng/100 µL ASAs concentration, which was significantly different from the positive control group (p ≤ 0.05). The antibody index % mean values were not significantly different at any of the samples collection weeks in compared to positive control values.

The ODs of the cutoff values of the serum samples obtained from Frankincense-treated TSA rats were measured. The results showed that the OD of the cutoff value in the 2^nd^ week was detected at the 1/132 serial dilution and 1/16 serial dilution for serum samples collected at the 4^th^, 6^th^, and 8^th^ weeks. The results revealed that at week 2, the serum of TSA rats treated with frankincense had an OD at first dilution (1/1) of 0.8865 ± 0.0505 and an ASAs concentration of 155.5 ± 1.45 ng/100 µL. At week 4, however, the OD at first dilution (1/1) was 0.616 ± 0.226, and the ASAs concentration was 106.4 ± 66.6 ng/100 µL. The results also showed that at week 6, the OD at first dilution (1/1) was 0.645 ± 0.225 and the ASAs concentration was 108.05 ± 57.95 ng/100 µL. In addition, in week 8, the OD at first dilution (1/1) was 0.58 ± 0.08, and ASAs concentration of 71.4 ± 17.7 ng/100 µL, which was significantly lower than the positive control group (p ≤ 001). The mean antibody index (%) was significantly decreased only at weeks 6 and 8 of treatment (p ≤ 0.001).

The ODs of the cutoff values of the serum samples obtained from secondary antibody-treated TSA rats were measured. The findings demonstrated that the cutoff value for the TSA rats treated with secondary antibodies was detected at the 1/8 serial dilution for weeks 2 and 4 and at the 1/16 serial dilution for weeks 6 and 8. The results showed that in the secondary antibody-treated group, the OD at first dilution (1/1) at week 2 was 0.487 ± 0.05 with an ASAs concentration of 48.15 ± 5.55 ng/100 µL, significantly decreased compared to the positive control group (p ≤ 0.001). However, at week 4, the OD at the first dilution (1/1) was 0.58 ± 0.08 with an ASAs concentration of 109.85 ± 56.15 ng/100 µL. In addition, the results revealed that at week 6, the OD at first dilution (1/1) was 0.494 ± 0.1, with 79.25 ± 21.75 ng/100 µL ASAs concentration significantly lower than the positive control group (p ≤ 0.01). Furthermore, at week 8, the OD at the first dilution (1/1) was 0.487 ± 0.05, with an ASAs concentration of 48.15 ± 5.5 ng/100 µL significantly reduced compared to the positive control group (p ≤ 0.001). The mean values of the antibody indexes (%) were significantly decreased in all weeks except for samples collected at week 4 (p ≤ 0.001).

### Testosterone levels assessment

As shown in Figure-4, the testosterone levels were determined for all treatment groups. Compared to the negative control group, the testosterone concentrations were significantly reduced in the positive control group (p ≤ 0.05) with the TSA rats treated with either AZA or DEX not significantly different from the positive control rats. However, the testosterone levels returned to normal in TSA rats treated with either Frankincense or secondary antibodies, as they were no different from the negative controls and significantly decreased when compared to the positive control (p ≤ 0.05).

### Histological examination

**Figure-5:**
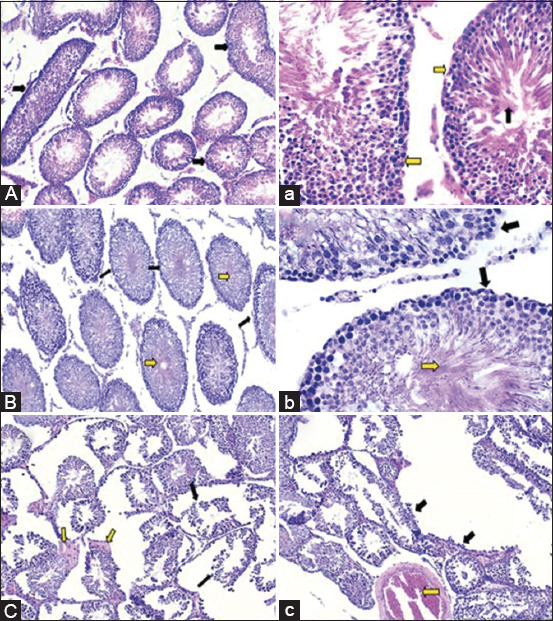
Haemotoxylin and Eosin-stained testicular tissues (100× and 400×) of controls and dexamethasone (DEX)-treated testicular sperm aspiration rats. (A and a) Testis of the negative control rats showed normal seminiferous tubules containing all spermatogenic cell stages (arrows) 100× which clarified with its high magnification at 400×. (B and b) Testis of the positive control rats displayed highly physiologically active seminiferous tubules where their lumen appeared completely occluded with sperm cells (yellow arrow). Furthermore, mild necrosis of some of the spermatogenic cells associated with folding and annulation in the basement membrane of some tubules (black arrows) 100× that displayed more clearly with its high magnification at 400×. (C and c) Testis of the DEX-treated group showed multifocal rupture and severe damage for large numbers of the seminiferous tubules (black arrows). Furthermore, moderate edema among some tubules was observed to be associated with severe dilatation and congestion of the testicular blood vessels (yellow arrows) 100×.

**Figure-6:**
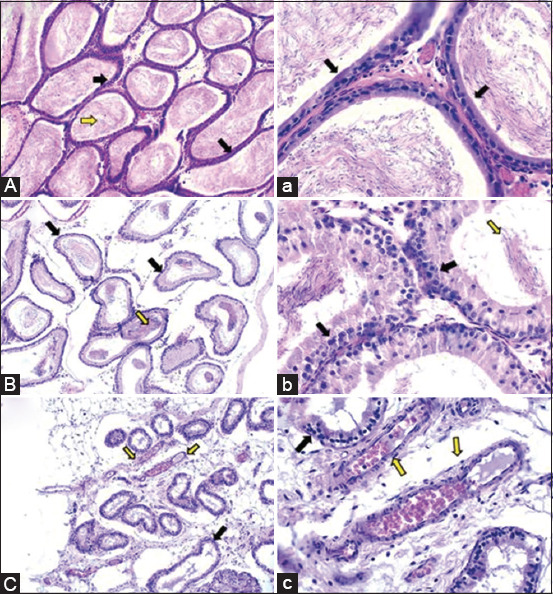
Haemotoxylin and Eosin-stained epididymal tissues (100× and 400×) of controls and dexamethasone (DEX)-treated testicular sperm aspiration rats. (A and a) Epididymis of the negative control rats displayed normal epididymal tubules which are completely field with the stored spermatozoa (yellow arrows) and lined with healthy intact pseudostratified columnar epithelium (black arrows). 100×, which also illustrated clearly at high magnification 400×. (B and b) Epididymis of the positive control rats exhibited normal epididymal tubules lined with normal intact pseudostratified columnar epithelium (black arrows). The tubules contain mild to moderate amount of the stored sperm cells (yellow arrows) 100×. At high magnification focal exfoliation was found in some areas of the lining epithelium (black star) 400×. (C and c) Epididymis of the DEX-treated group displayed a decrease in the existence of the epididymal sperms with mild degeneration in their lining epithelium (black arrows). Furthermore, mild congestion in the testicular blood vessels was observed (yellow arrows) 100× which became clearer in the high magnification 400×.

In the negative control rats, the seminiferous tubules in the testes were normal, demonstrating the existence of several spermatogenic cell stages such as spermatogonial mother cells, primary and secondary spermatocytes, and spermatids occluding the majority of the lumen of seminiferous tubules (Figures-5A and a). The epididymis showed typical epididymal tubules that were bordered with healthy, undamaged pseudo-stratified columnar epithelium and filled completely with seminal fluid (Figures-6A and a).

Histological examination of the positive control TSA rats’ testicular tissue showed the presence of distinct phases of spermatogenic cells completely occluded the tubular lumen resulting in highly physiologically active seminiferous tubules. Some tubules showed modest spermatogenic cell necrosis along with folding and annulation in the basement membrane (Figures-5B and b). The epididymis of rats in the positive group exhibited normal epididymal tubules lined with normal intact pseudo-stratified columnar epithelium. The tubular lumen contains mild to moderate amount of sperm cells (Figures-6B and b).

The primary histological findings in the testicular tissue of the DEX-treated group were multifocal rupture and severe destruction to a significant portion of the seminiferous tubules. In addition, several tubules showed moderate edema (Figures-5C and c). Other testes showed desquamation of the spermatogenic cells of the seminiferous tubules in addition to mild peri-tubular edematous fluid infiltration. Also showed atrophy and shrinkage in several seminiferous tubules associated with congestion in the testicular blood arteries (Figures-5C and c). The epididymis tissue exhibited a reduction in the number of epididymal sperms and a modest degradation in their lining epithelium. In addition, there was minor congestion in the testicular blood vessels (Figures-6C and c).

**Figure-7:**
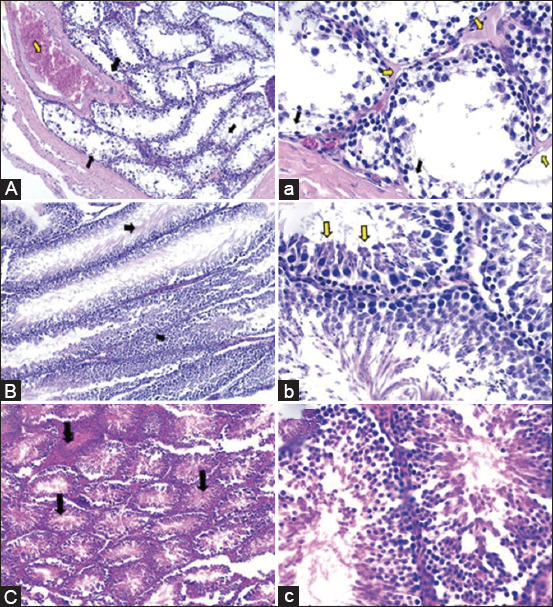
Haemotoxylin and Eosin-stained testicular tissues (100× and 400×) of azathioprine (AZA), frankincense, or secondary antibody-treated testicular sperm aspiration rats. (A and a) Testis of the AZA-treated rats showed degeneration, desquamation, and necrosis of the spermatogenic cells in several seminiferous tubules (black arrows). In addition, severe congestion of the testicular blood vessels was also observed 100×, and in the higher magnification, mild to moderate intertubular edematous fluid infiltration was found (yellow arrows) 400×. (B and b) Testis of the frankincense-treated group showed high spermatogenic activity and the seminiferous tubules impacted with the different stages of spermatogenic cells leaving a narrow lumen (black arrows) 100×. The higher magnification illustrated intact and active Sertoli cells (yellow arrows) 400×. (C and c) Testis of the secondary antibody-treated rats showed highly active seminiferous tubules characterized by the existence of the different stages of the spermatogenic cells which nearly occlude the tubular lumen (black arrows) 100×. The higher magnification clarified the existence of the healthy intact Sertoli cells (yellow arrows) 400×.

**Figure-8:**
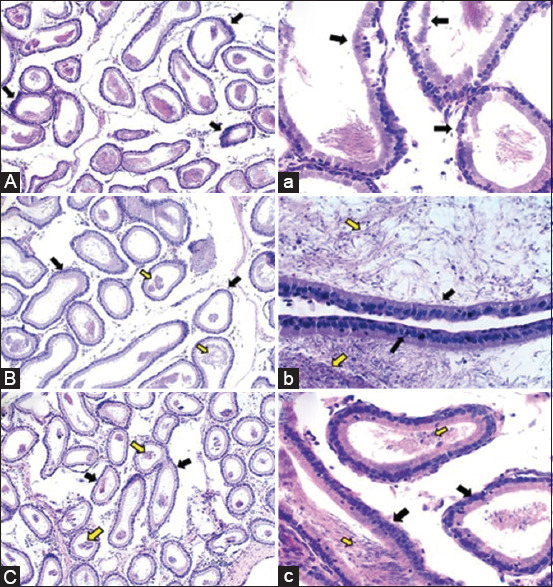
Haemotoxylin and Eosin-stained epididymal tissues (100× and 400×) of Azathioprine (AZA), frankincense, or secondary antibody-treated testicular sperm aspiration rats. (A) Epididymis of the AZA-treated rats showed multifocal hyperplasia in the lining epithelium of several epididymal tubules (arrows) 100×. (a) Epididymis of the same group clarified desquamation and necrosis in the lining epithelium of the epididymal tubules (arrows) 400×. (B and b) Epididymis of the frankincense-treated rat showed normal epididymal tubules lined with healthy intact pseudostratified columnar epithelium (black arrows). The epididymal tubules contain a moderate amount of sperm cells 100× that clarified obviously in its higher magnification 400×. (C and c) Epididymis of the secondary antibody-treated rat exhibited normal epididymal tubules lined with normal intact pseudostratified columnar epithelium (black arrows). Furthermore, the epididymal tubules are moderately filled with stored sperm cells (yellow arrows) 100×. These findings are illustrated obviously in its higher magnification of 400×.

The spermatogenic cells in various seminiferous tubules of the testicular tissues of the AZA-treated rats were degenerated, desquamated, and necrosed. Along with modest to moderate inter-tubular edematous fluid infiltration, there was also severe congestion in the testicular blood vessels within the seminiferous tubules and in the tunica albogenia covering the testis (Figures-7A and a). Significant atrophy and shrinkage were seen in other seminiferous tubules (Figures-7A and a). The lining epithelium of the epididymal tubules showed multifocal hyperplasia in numerous tubules (Figures-8A and a).

The frankincense-treated group showed no marked histopathological changes, and the testicular tissue displayed a high spermatogenic activity as the seminiferous tubules were impacted by different stages of the spermatogenic cells, leaving a narrow tubular lumen. Intact and active Sertoli cells were also seen. The interstitial well-developed polyhedral Leydig cells were also detected (Figures-7B and b). Normal epididymal tubules lined with healthy intact pseudo-stratified columnar epithelium and the tubular lumen containing a moderate amount of the stored sperm cells were noticed in this group (Figures-8B and b).

The examined tissue samples in the secondary antibody-treated rats revealed a normal histological picture in general; with some areas showing histopathological damage. The testicular tissue showed active seminiferous tubules characterized by the presence of different stages of the spermatogenic cells that nearly occlude the tubular lumen associated with the presence of healthy intact Sertoli cells (Figures-7C and c). Normal epididymal tubules lined with normal intact pseudo-stratified columnar epithelium were observed. Furthermore, the epididymal tubules are moderately filled with stored sperm cells (Figures-8C and c).

## Discussion

The main male reproductive organ, the testis, produces spermatozoa and testosterone. When the immune system is unable to discriminate between sperms that are self-antigens, and those that are not, autoimmunity infertility results, and ASAs are released to target one’s own sperms. However, there are debates over the clinical significance of detecting ASAs in serum and seminal plasma, and it is yet unknown why ASAs are tested. Immunosuppressive medications such as corticosteroids are commonly used to treat autoimmune disorders [11]. If the test revealed ASAs, treatment with modest dosages can be suggested [12]. In the current investigation, several therapeutic methods, including DEX, AZA, frankincense, and produced secondary antibodies with established efficacy were utilized.

Testicular sperm aspiration has been utilized to extract sperm from humans and several animal species, including rams, and rat models [18–20]. Prithiviraj *et al*. [33] and Shufaro *et al*. [34] both demonstrated that biopsies significantly elevate and produce ASAs. As a result, TSA was utilized in the current study as a rat model for ASAs production.

Our data indicated that the positive control rats’ testosterone levels were significantly lower than those of the negative control group. This finding contrasts with the study on rats by Barroso *et al*. [35], who did not find any change in testosterone levels, and it is consistent with human studies conducted following TSA [23, 36]. According to our findings, the ASAs titer of the positive control TSA rats was 1/512 at week 2, 1/64 at week 4, and 1/128 at weeks 6 and 8, with a mean antibody index (%) of 24.97. During each of the examined weeks, the positive controls’ ASAs levels were significantly higher than those of the negative controls. The testicular tissue of the positive control TSA rats underwent histological examination, which revealed little spermatogenic cell necrosis as well as folding and annulation in the basement membrane. Sperm cells were present in minor to moderate levels in the epididymis’ tubular lumen. These results substantially support the previous findings of Prithiviraj *et al*. [33]. After sperm extraction, men’s serum samples contained ASAs of the IgG class, according to Wood *et al*. [21]. Following the biopsy, hematomas and scar tissue were observed in human testicles [22]. Humans have been reported to be at risk for postoperative fibrosis, according to studies by Schlegel and Su [22] and Yildirim *et al*. [37].

The testosterone levels in TSA rats treated with AZA were unchanged when compared to the positive control group. According to our findings, the AZA-treated TSA group’s cutoff value OD was seen at serial dilutions of 1/8 and 1/64 for weeks 2 and 4, respectively, and at a serial dilution of 1/16 for weeks 6 and 8. Our results have also shown that the OD at first dilution (1/1), ASAs concentrations, and the mean of the antibody index values (%) in the 2^nd^ and 4^th^ weeks was significantly lower than the positive control group. However, the ASAs indexes tend to be higher in the 6^th^ and 8^th^ weeks suggesting an earlier effect for AZA on ASAs. The testicular tissues of the rats treated with AZA had spermatogenic cells that were degenerating, desquamating, and necrotic. In addition to the mild to moderate inter-tubular edematous fluid infiltration, testicular blood vessels within the seminiferous tubules and the tunica albuginea covering the testis were severely congested. There was noticeable atrophy and shrinkage in other seminiferous tubules. Numerous tubules of the epididymal tubules’ lining epithelium displayed multifocal hyperplasia as demonstrated by Chavez-Alvarez *et al*. [13]. AZA inhibits DNA synthesis in cells that proliferate rapidly, such as T and B lymphocytes [38]. AZA harms fertility, causing damage to the testicular structure and disrupting testicular integrity in mice, as has been previously reported [25]. AZA has been demonstrated to reduce immunity and lowers the number of killer cells, according to Honkila *et al*. [39].

As they were identical to the negative controls and much lower when compared to the positive controls, the results revealed that testosterone levels in TSA rats treated with frankincense reverted to normal. The OD of the cutoff value in the 2^nd^ week was detected at the 1/132 serial dilution and 1/16 serial dilution for serum samples collected at the 4^th^, 6^th^, and 8^th^ weeks. In addition, in week 8, the ASAs concentration was significantly lower than the positive control group. The mean antibody index values (%) were significantly decreased only at week 6 and 8 of treatment, suggesting the late efficacy of frankincense on ASAs.

The frankincense-treated group did not exhibit any noticeable histological abnormalities. Testicular tissue examination revealed a high level of spermatogenic activity as the seminiferous tubules were influenced by various phases of the spermatogenic cells, leaving a restricted tubular lumen. Sertoli cells that were both intact and active were observed. In this group, normal epididymal tubules were seen, bordered by healthy, undamaged pseudo-stratified columnar epithelium, and the tubular lumen contained a modest amount of the stored sperm cells. Recent research indicated that frankincense regulates immune cells from the innate and acquired immune systems, and inhibits leukotriene synthesis, oxidative stress, and leukotriene production [15]. Frankincense was used in the past as an anti-inflammatory drug, particularly for arthritis [14, 26]. These studies also reported that the weight of the prostate, seminal vesicles, and epididymis dramatically increased in their work on male fertility. In addition, the testosterone and FSH levels as well as the number of sperm and their motility were increased because of the presence of more primary and secondary sperm cells.

The testosterone levels in TSA rats treated with DEX were not different from the positive control group compared to the negative control group. The findings showed that at week 2, serial dilution 1/128 was the threshold at which the cutoff value’s ODs were found in the serum samples. The cutoff value was detected at the 1/64 serial dilution for the 4^th^ and 6^th^ weeks of therapy and the 1/32 serial dilution for the 8^th^ week of treatment. In addition, the concentration of ASAs was significantly lower than the positive control group at week 8. The antibody index% mean values were not statistically different from the positive control values at any of the sample collection week.

The main histopathological findings in the testicular tissue of the DEX-treated group were multifocal rupture and severe damage to a considerable section of the seminiferous tubules. Several tubules furthermore exhibited mild edema. On the other hand, some rats’ testes displayed atrophy and shrinkage in several seminiferous tubules linked to congestion in the testicular blood vessels. The tissue of the epididymis showed a decrease in the number of epididymal sperms and a modest deterioration of their lining epithelium. There have recently been conflicting studies favoring and opposing treating ASAs with corticosteroids, according to Barz, a member of the Center for Infertility Treatment and Embryo Research [11]. Contrarily, Bubanovic *et al*. [40] found that corticosteroids have a beneficial impact on the immune system and a direct impact on immunoglobulin responses, making DEX a safe therapy for ASAs infertility in males. According to Lu *et al*. [41], there is no therapy for ASAs (autoimmune illnesses), but in general, the premise of treatment relies on suppressing the production of antibodies. Dexamethasone improves patient assurance by repressing antibody levels, which in turn improves spermatozoon parameters and strengthens fertility. Corticosteroids are often employed in the treatment of inflammatory and autoimmune illnesses as well as in organ transplantation, as demonstrated by Leroy *et al*. [42]; however, the adverse effects may last for some time.

The results of the secondary antibody therapy showed that the testosterone levels in TSA rats reverted to normal, as they were identical to the negative controls and much lower than the positive control. For weeks 2, 4, 6, and 8, the cutoff value for the TSA rats treated with secondary antibodies was discovered at the 1/8 serial dilution. The findings demonstrated that during all study weeks, the ASAs concentration was significantly lower in the secondary antibody-treated group than in the positive control group. Except for week 4, samples had a substantial decline in the mean antibody indices (%) compared to the positive group. The tissue samples from the secondary antibody-treated rats indicated a generally normal histological appearance. The testicular tissue had active seminiferous tubules, which are defined by the presence of spermatogenic cells at various stages that almost completely occlude the tubular lumen in association with the presence of normal, intact Sertoli cells. In addition, a little amount of sperm cells was retained in the epididymal tubules.

The immune system can produce millions of distinct antibodies that can specifically recognize and target other antibodies that are aware of their molecular structures. The immune system can be manipulated by taking advantage of these interactions, also known as idiotype-anti-idiotype responses, which may have significant effects on how certain autoimmune illnesses can be treated. Idiotype is the previous name for the distinct antigenic determinant on the primary antibody. The term “anti-idiotype” was created to describe the secondary antibody produced in reaction to the idiotype [16]. According to Pan *et al*. [43], secondary antibodies have recently gained popularity as an alternative type of immunotherapy since they can target autoantibodies selectively, have low toxicity and adverse effects, and perhaps provide long-lasting immunity. Anti-idiotypic antibodies are now being researched as potential novel therapeutics for cancer, autoimmune disorders, and some other diseases. In their review, Stanova *et al*. [17] made the speculative claim that a secondary antibody would be able to change an antigen’s biological action. According to Naz *et al*. [44], secondary antibodies can counteract the anti-fertilization antigen FA-1 antibodies’ ability to prevent fertilization when sperm enters the egg. In addition, it may stop sperm antibodies from adhering to the sperm’s surface.

In this study, rabbits were utilized for the generation of therapeutic secondary antibodies because rabbits may produce a more robust, high-affinity repertoire of antibodies than other laboratory animals such as rats and mice. According to Großerichter-Wagenera *et al*. [16], rabbits have long been utilized as a dependable source of polyclonal antibodies and exhibit stronger antibody responses than mice. They showed that generation of antibodies as a response to antigen-specific antibodies is known as anti-idiotype responses, presumably because the idiotype region is the sole “foreign” section of the antibody molecule.

## Conclusion

This study produced realistic treatment recommendations despite the inconsistent outcomes of the various treatments used, which are caused by a variety of factors, including the complexity of the mechanisms involved in ASAs development, the technical methods used to measure ASAs, and the lack of agreement over the appropriate thresholds to be utilized. It will be feasible to collect reliable, comparable data that can further our understanding. In addition, there has been little progress in developing viable therapies; immunotherapy is one promising area for future studies. Finally, caution should be applied when drawing conclusions from experimental models to address clinical needs.

## Authors’ Contributions

AAZ and SMA: Designed and supervised the study. FSAld, FSA, and AAZ: Performed the TSA procedure and developed the ASAs ELISA. AAM: Performed the histological examination. KAA: Quantified the testosterone levels. AAZ and SMA: Analyzed the data and wrote the manuscript. All authors have read, reviewed, and approved the final manuscript.
